# Performance evaluation of thin film silicon solar cell based on dual diffraction grating

**DOI:** 10.1186/1556-276X-9-688

**Published:** 2014-12-19

**Authors:** Raghvendra Sarvjeet Dubey, Sigamani Saravanan, Sivaperuman Kalainathan

**Affiliations:** Advanced Research Laboratory for Nanomaterials and Devices, Department of Nanotechnology, Swarnandhra College of Engineering and Technology, Seetharampuram, Narsapur, Andhra Pradesh India; Centre for Crystal Growth, School of Advanced Sciences, VIT University, Vellore, Tamilnadu India

**Keywords:** Dual grating, Absorption, Short-circuit current, Thin film solar cells

## Abstract

Light-trapping structures are more demanding for optimal light absorption in thin film silicon solar cells. Accordingly, new design engineering of solar cells has been emphasized and found to be effective to achieve improved performance. This paper deals with a design of thin film silicon solar cells and explores the influence of bottom grating and combination of top and bottom (dual) grating as a part of back reflector with a distributed Bragg reflector (DBR). Use of metal layer as a part of back reflector has found to be promising for minimum requirement of DBR pairs. The effect of grating and anti-reflection coating thicknesses are also investigated for absorption enhancement. With optimization, high performance has been achieved from dual grating-based solar cell with a relative enhancement in short-circuit current approximately 68% while it was approximately 55% in case of bottom grating-based solar cell. Our designing efforts show enhanced absorption of light in UV and infrared part of solar spectrum.

## Background

Nowadays, non-conventional energy sources are demanding alternatives to overcome the problem of power scarcity at worldwide. This need has generated a huge scope of research in fundamental and advanced branches of science and technology. Severe work on silicon thin film solar cells including design and fabrication has been focused by scientific community. Silicon technology is well known which is safe, non-toxic, and cheaper for thin film solar cells; however, weak absorption in longer wavelength is a major issue which needed to be attained to a maximum possibility. To overcome this problem, new design engineering of solar devices are to be spotlighted which includes an efficient light-trapping structure. For efficient light-trapping structures, one-dimensional photonic crystal also known as distributed Bragg reflector and diffraction grating are the components of bottom reflector and have been explored for thin film silicon solar cells. Distributed Bragg reflector provides total internal reflection of longer wavelength light that passes through absorbing layer of solar cell; however, diffraction grating diffracts and scatters the light. Diffraction grating can increase the light absorption by a factor of 4n^2^ (*n* is medium's refractive index) through diffraction of the light at higher angles so that light can propagate through medium or couple the guided lights. The combination of both DBR and grating as back reflectors enforces the light towards active region and as a result enhances the absorption.

Several ideas of light trapping in solar cells have been reported by the researchers such as random or periodic pyramids, gratings, plasmonic nanoparticles, one-dimensional photonic crystals, etc [1–4]. Zeng et al. have demonstrated a combined effect of one-dimensional photonic crystal as a distributed Bragg reflector and diffraction grating on the performance of thin film solar cells. They have observed enhanced light absorption in active region due to high reflectivity and large angle diffraction. Experimentally, short-circuit current density was observed to be increased by 19% as comparison to simulated results [5]. Rao et al. have presented a modeling of a solar cell based on silicon diffraction grating [2]. A solar cell structure with 500 nm period and 150 nm depth has produced 76.5% enhancement of short-circuit current density. They have concluded that the positioning of grating on the rear surface can reduce the short wavelength losses. Further, the use of high refractive index material (silicon) can result in strong diffraction while light absorbed in the grating pillar can yield better performance of the device. Kuo et al. have demonstrated an amorphous silicon-based solar cell with a backside distributed Bragg reflector [6]. They have suggested using of more than one DBR structure as a part of back reflector in order to utilize wider bandwidth of solar spectrum. A solar cell with a combination of three DBR pairs has produced optimal quantum efficiency due to transmission of shorter wavelength and reflection of longer wavelength of light. Gjessing et al. have presented a numerical investigation of a light-trapping structure consisting of a two-dimensional back diffraction grating in combination with an aluminum reflector [7]. An enhancement in short-circuit current density from 30.4 mA/cm^2^ to 35.5 mA/cm^2^ has been achieved from 20 μm thick silicon solar cell. This enhancement in current density has been attributed to the increased path length due to in-coupling of light with decreased parasitic absorption in aluminum medium due to a spacer layer used. Recently, there is an intensive research that can be seen on multiple material-based grating structure with a combination of DBR. Abass et al. have numerically presented a study of complex dual interface grating system for thin film silicon solar cells which is just the combination of a plasmonic grating at the back side and a dielectric grating at the front side of the cell [8]. Such structures have shown a strong coupling of higher-order guided modes in addition to coupling of lower-order modes. Further, combination of blazing and dual interface grating structures were observed to be more accessible modes with a strong coupling efficiency which ultimately enhanced the light absorption. Chriki et al. have presented the solar cell architecture design with the use of two periodic layers of metallic and dielectric grating and analyzed that both layers can couple the incident light to photonic and plasmonic modes; as a result, enhanced absorption can be assured [9]. The importance of the relative shift between these two gratings has been explored and shown to be crucial in enhancing the current density via the mechanism of coupling to dark modes providing additional absorption. Schuster et al. have reported a simple layer transfer fabrication technique to enhance light trapping which allows independent patterning of thin film at both the sides. The fabricated dual grating structure showed an improvement over single grating pattern either on the top or bottom of the film [10]. Zhao et al. have demonstrated a design of solar cell with an indium tin oxide diffraction grating, a DBR of a-Si:H/ITO and an Ag reflector [11]. With the use of metal reflector, they have observed 69% and 72% weighted absorptance of solar cell in cases 4 and 8 pairs of a-Si:H/ITO DBR, respectively. It is claimed that the use of metal reflector is helpful to trap light in a better way with reduced number of DBR pairs and hence makes easy fabrication. Mutitu et al. have presented a light-trapping design which can be applied to stand alone and multiple junction thin film silicon solar cells [12]. This design includes diffraction grating and one-dimensional photonic crystals as band pass filters that reflect short light wavelengths and transmit longer wavelengths at the interface between two adjacent cells. Enhanced short-circuit current approximately 30.25 mA/cm^2^ was achieved with 5 μm cell thickness solar cell based on top and bottom triangular grating with a DBR.

In this paper, we present a design of thin film silicon solar cell by employing FDTD method and have found promising for absorption enhancement in UV and infrared part of solar spectrum. With the influence of bottom and top grating, it is observed that the bottom grating enhances short-circuit current by *55%* whereas combination of top and bottom (dual) grating yields *68%* due to enhanced absorption. In the second section, simulation approach is presented and simulated results are discussed in the third section. Finally, the fourth section concludes the paper.

## Methods

**Figure 1:**
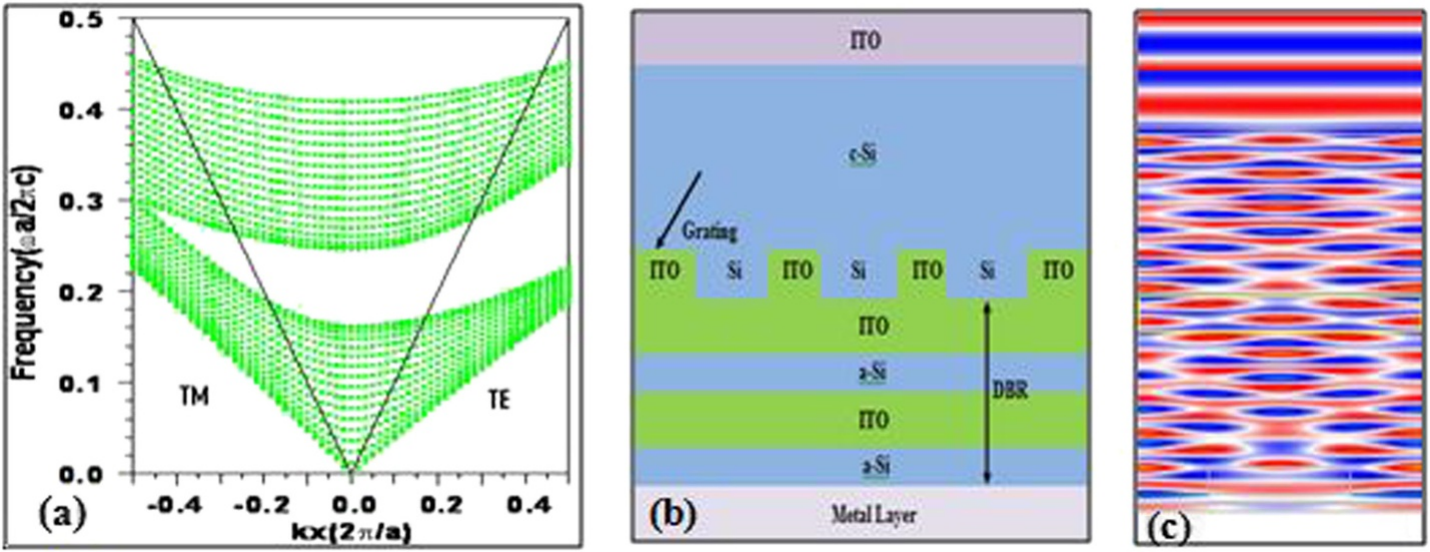
**Photonic band structure (a) and schematic diagram of designed solar cell structure (b) and electric field distribution profile (c).**

Distributed Bragg reflector is composed of low and high refractive index layers arranged periodically in one direction. Plane wave method is one of the well-known techniques used to calculate band structure of photonic crystals. Here, we have designed a DBR which is composed of alternate layers of indium tin oxide and amorphous silicon (ITO/a-Si) respectively with their periodicity in x direction. For simulation, we have used plane wave method by applying perfectly matched layer boundary condition to y and z directions. Figure 1a shows band diagram of a DBR for both TE and TM polarizations. The considered values of refractive index are *n*_a-Si_ = 3.6 and *n*_ITO_ = 1.85, thickness t_a-Si_ = 56 nm and *t*_IT0_ = 108 nm, and lattice constant *Ʌ* = *t*_a-Si_ *+ t*_ITO_ *=* 164 nm with center wavelength approximately 800 nm. At normal incidence, a complete photonic band gap is obtained at frequency *0.162* to *0.242 (in the units of ωa/2πc)* and so longer wavelength light can be trapped and enforced towards active silicon region after total internal/Bragg reflection. This designed DBR is further incorporated into a design of 5 μm thickness solar cell in between grating and metal layer as shown in Figure 1b. This solar cell consists of indium tin oxide-antireflection coating layer (ITO), crystalline silicon (C-si)-active region, and a back reflector composed of diffraction grating, DBR, and metal layer.Use of DBR as a part back reflector doubles path length of longer wavelength photons due to evanescent decay in DBR and enforcing them into active region after total internal or Bragg reflections. Figure 1c shows electric field profile in designed solar cell. Grating diffracts longer wavelength light of single passed from active silicon region as short wavelength light is already being absorbed before it crosses active region. In simple words, once the light made incident onto the solar cell, the shorter wavelength light is being absorbed; however, longer wavelength light is passed through active silicon region and reaches to DBR where it decays evanescently and coming back after reflections. The occurrence of diffraction and scattering prolongs the optical path length in the absorbing layer which supports coupling of light and hence enhancement of solar cell performance can be expected.

## Results and discussion

**Figure 2:**
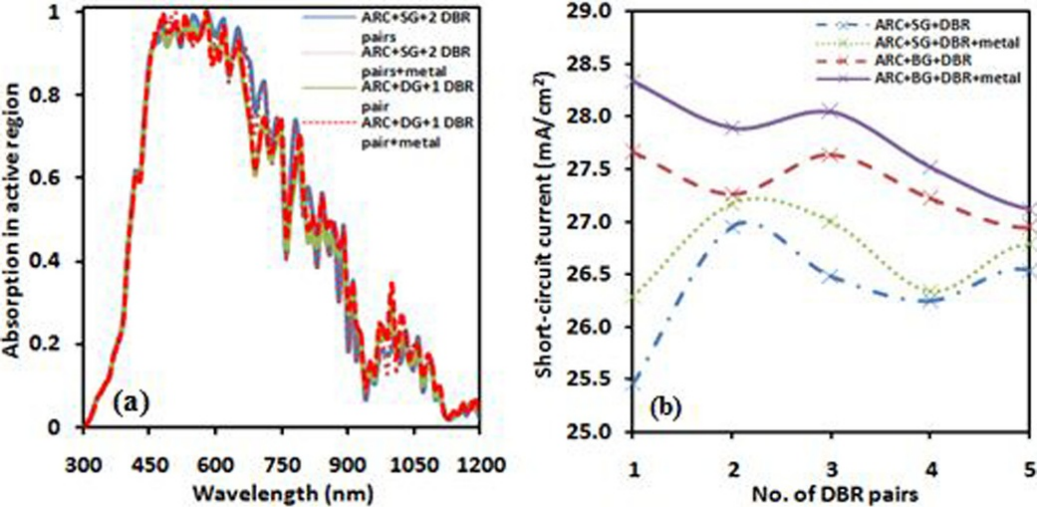
**Absorption spectra.** Absorption spectra of solar cell designs A, B, C, and D **(a)** and short-circuit current as a function of DBR pairs **(b)**, respectively.

In this work, we have explored the analysis of single layer (SG) (refer to Figure 1b), double layer (DG), and dual grating-based solar cells (refer to inset Figure 4b). We have designed four solar cells named as cell A (ARC + SG + 2 DBR), cell B (ARC + SG + 2 DBR + metal), cell C (ARC + DG + 1 DBR), and cell D (ARC + DG + 1 DBR + metal). Figure 2a shows absorption spectra of four solar cells A, B, C, and D. The curve of solar cell A depicts overlapped absorption curves with others at shorter wavelength but enhanced absorption beyond approximately *510 nm*. Further, improved absorption can be noticed at approximately *850* and *1060 nm*. By increasing number of DBR, an enhancement of light absorption was observed (not shown here) but it was maximum for two pairs of DBR-based solar cell. For cell B, use of a metal layer has given a similar trend in curve but with enhanced absorption as compared to the case of without metal layer. By comparing curves of cells A and B, we can observe enhanced absorption from cell B in wavelength range *507* to *1060 nm.* The obtained short-circuit current are for cell A approximately *26.95* and cell B approximately *27.17 mA/cm*^*2*^, respectively. Absorption curves of cells C ad D show distinct results when single grating is replaced with double grating. By comparing curves of cells A and B, we can observe an enhancement in absorption; however, it is dominant in infrared region of incident solar spectrum. It is noteworthy that one DBR pair with double grating combination produces short-circuit current up to approximately *27.65 mA/cm*^*2*^ whereas it was *26.95 mA/cm*^*2*^ for two DBR pairs with a single grating combination. Further, the use of a metal layer (cell D) has improved the performance of solar cell in the wavelength approximately *510* to *1060 nm*.

Among four above discussed solar cells, double grating with one DBR pair and metal layer-based solar cell (cell D) gave *46%* relative enhancement in short-circuit whereas it is *40%* for cell B. Figure 2b shows comparison of short-circuit current of best performed solar cells as a function of number of DBR pairs. The short-circuit current is found to be maximum approximately *26.95* and *27.17 mA/cm*^*2*^ for the cells A and B with two DBR pairs; however, cells C and D show enhancement in current approximately *27.65* and *28.33 mA/cm*^*2*^ with the use of only one DBR pair. The use of double grating as a part of back reflector yields enhanced performance of the devices whereas metal layer plays a promising role to reduce the requirement of DBR pairs and hence, makes easy fabrication of solar cell.

**Figure 3:**
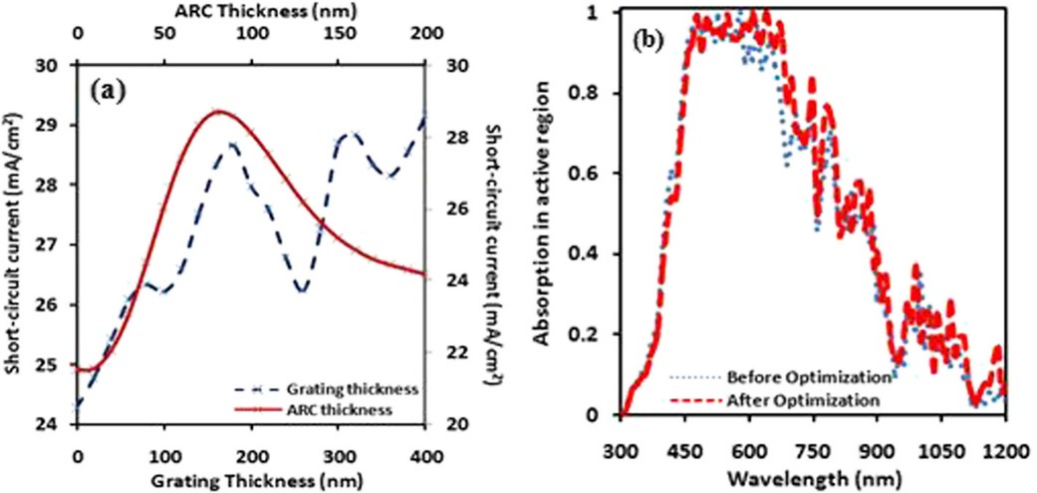
**Short-circuit current of cell D and comparison of absorption spectra.** Short-circuit current of cell D **(a)** as a function of anti-reflection coating and grating thickness and absorption spectra **(b)** before and after optimization, respectively.

The best performed solar cell D is again modeled for the optimization of anti-reflection coating layer thickness *(t*_arc_*)* and grating thickness *(G*_*t*_*)* which is shown in Figure 3a. The optimal short-circuit current approximately *28.6 mA/cm*^*2*^ is obtained *at t*_arc_ *=* 80 nm and further it is found to be improved approximately *29.2 mA/cm*^*2*^*at G*_*t*_ *=* 400 nm*.* These optimized values of *t*_arc_ and *G*_*t*_ were used to remodel the solar cell and observed an enhancement in absorption. Figure 3b shows the absorption spectra of optimized and without optimized solar cells as a function of incident solar spectrum. We can observe the broadening of light absorption curve in the range *470* to *725* nm and further enhanced absorption in infrared part of wavelength.

**Figure 4:**
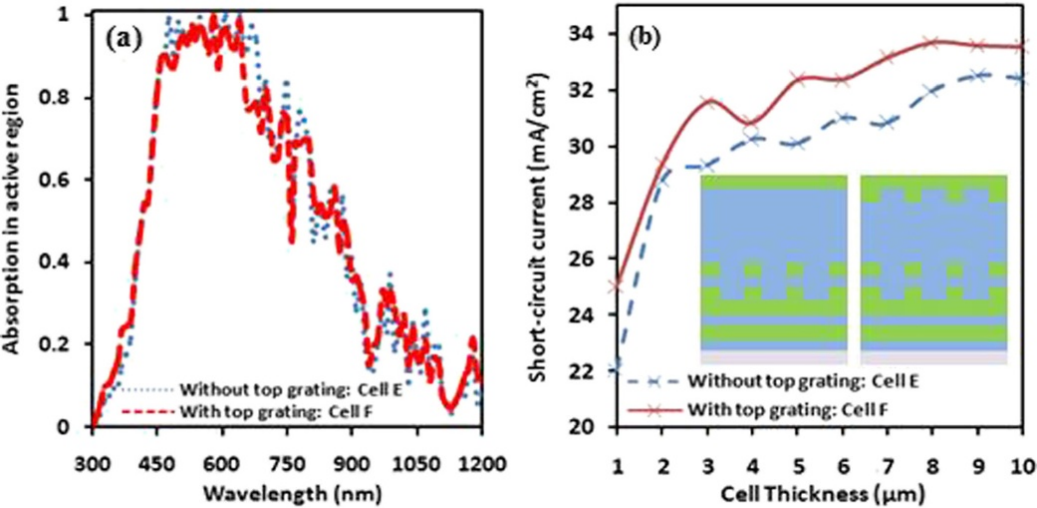
**Absorption spectra and short-circuit current.** Absorption spectra as a function of incident spectrum **(a)** and short-circuit current **(b)** as a function of cell thickness of cells E and F, respectively.

To observe the improved performance of the above designed solar cell based on double grating back reflector, we have placed one more grating at the top of active silicon region. Figure 4a shows the comparison of absorption in silicon active region of solar cells without top grating (named cell E) and with top and bottom grating/dual grating (named cell F). An improvement in absorption can be observed for shorter wavelength as well as longer wavelength from approximately *780* to *1200 nm*. This shows an efficient harvesting of light into solar cell based on dual grating. The short-circuit current (Jsc) obtained from cells E and F as a function of cell thickness is plotted in Figure 4b. The designed solar cell structures with and without top grating is shown in the inset of Figure 4b. Both curves depict as usual effect of cell thickness while dual grating based solar cell is found to be more efficient design with improved short-circuit current as comparison to cell E. The optimal obtained short-circuit current of cell E and F are approximately *30* and *31 mA/cm*^*2*^, respectively. Remarkably, the use of top grating showed diffraction of shorter wavelength (UV) whereas longer wavelength (IR) trapping is observed by bottom reflector.

## Conclusions

We have designed and analyzed the performance of solar cells by considering various parameters. It is observed that the combination of metal layer at the bottom with grating and DBR reduces the requirement of DBR pairs. The double bottom grating-based solar cell showed better performance against to single grating. Our results showed that the use of bottom double grating is helpful to diffract longer wavelength whereas top grating is found to be a promising one to diffract shorter wavelength of solar spectrum. A relative enhancement in short-circuit current approximately 68% and 55% is achieved with dual grating and double bottom grating-based solar cells, respectively. This presented designing of solar cell is helpful to trap UV and infrared part of solar spectrum whereas fabrication challenges remain.
